# The Diagnostic Value of Cerebrospinal Fluid Neurogranin in Neurodegenerative Diseases

**DOI:** 10.3390/ijms252413578

**Published:** 2024-12-19

**Authors:** Daria Krawczuk, Piotr Mroczko, Izabela Winkel, Barbara Mroczko

**Affiliations:** 1 Department of Neurodegeneration Diagnostics, Medical University of Bialystok, Waszyngtona 15A, 15-269 Białystok, Poland; daria.krawczuk@sd.umb.edu.pl; 2 Faculty of Law, University of Bialystok, Mickiewicza 1, 15-213 Białystok, Poland; mroczkopiotr@gmail.com; 3 Dementia Disorders Centre, Medical University of Wroclaw, 50-425 Ścinawa, Poland; i.winkel@me.com; 4 Department of Biochemical Diagnostics, Medical University of Bialystok, Waszyngtona 15A, 15-269 Białystok, Poland

**Keywords:** neurogranin, biomarkers, neurodegeneration, Alzheimer’s disease, Parkinson’s disease, Creutzfeldt–Jacob’s disease

## Abstract

Synaptic pathology is crucial in neurodegenerative diseases (NDs), and numerous studies show a correlation between synaptic proteins and the rate of cognitive decline in Alzheimer’s disease, Parkinson’s disease, dementia, and Creutzfeldt–Jacob’s disease. Due to the fact that altered synaptic function is considered a core feature of the pathophysiology of neurodegenerative disorders, synaptic proteins, such as neurogranin, may serve as a biomarker of these diseases. Neurogranin is a postsynaptic protein located in the cell bodies and dendrites of neurons, foremost in the cerebral cortex, hippocampus, and striatum. It has been established that neurogranin is involved in synaptic plasticity and long-term potentiation. Literature data indicate that cerebrospinal fluid neurogranin may be useful as a biomarker for more accurate diagnosis and prognosis of neurodegenerative diseases. In this review, the diagnostic value of cerebrospinal fluid neurogranin in most common neurodegenerative diseases is examined.

## 1. Introduction

**Figure 1:**
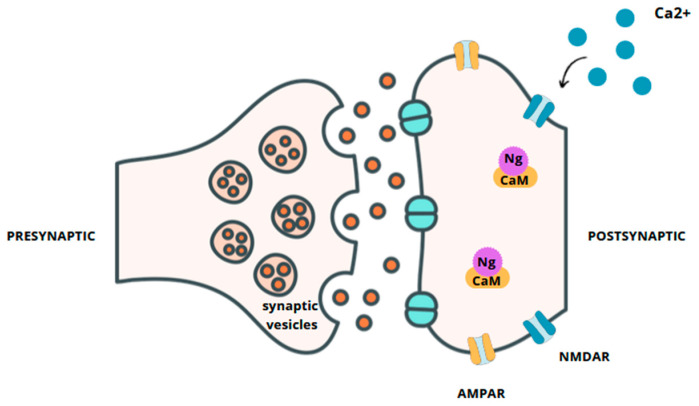
Localization of neurogranin.

Neurogranin (Ng) is a calmodulin-binding protein expressed in the cerebral cortex, hippocampus, amygdala, and striatum, particularly in postsynaptic neurons [1]. Although neurogranin is considered neuron-specific, it is also expressed in small amounts in the lungs, spleen, and bone marrow. In neurons, it is found concentrated at dendritic spines where it participates in synaptic signaling events through the regulation of calmodulin (CaM) availability [2]. Neurogranin binds to CaM under conditions of low Ca^2+^ concentration or in the absence of Ca^2+^, whereas it rapidly releases CaM in response to large amounts of Ca^2+^ [3]. The specific binding site for Ng in CaM is primarily located within the IQ motif of Ng, which is crucial for its interaction with CaM [2]. Ng enhances the postsynaptic sensitivity and increases synaptic strength in an activity- and the N-methyl-D-aspartate receptor (NMDAR)-dependent manner. In CA1 hippocampal neurons, synaptic NMDAR activation causes a local, fast Ca^2+^ increase in dendritic spines, allowing CaM to activate downstream effectors and resulting in long-term potentiation (LTP) [4]. Furthermore, long-term blockade of NMDAR significantly decreases Ng levels, thus indicating that Ng is regulated by synaptic activity and promotes synaptogenesis [5]. One of the factors modulating LTP is the Ca^2+^/calmodulin signaling pathway, which regulates synaptic enhancement through calcium/calmodulin-dependent protein kinase II (CaMKII), protein kinase C (PKC), and synaptic proteins activity [6]. In particular, Ng phosphorylation increases after LTP induction and decreases after long-term depression (LTD) induction. Furthermore, progressively increased Ng phosphorylation promotes sustained activation of synaptic transmission [2]. It is speculated that the role of Ng phosphorylation is to prevent the recapture of CaM when intracellular Ca^2+^ returns to basal levels. PKC γ has been shown to be the isoform involved in the in vivo phosphorylation of Ng. Since PKC γ needs Ca^2+^ and digliceryde (DAG) for activation, only those stimuli capable of activating ionotropic and metabotropic receptors simultaneously could notably increase Ng phosphorylation. Thus, Ng phosphorylation, progressively increased by higher-frequency stimuli, would promote sustained activation of synaptic transmission [7]. Figure 1 presents the localization of neurogranin and its relationship with calmodulin.

Age-related changes in post-synaptic Ng distribution in selected brain areas, particularly in the hippocampus, may contribute to altered Ca^2+^/calmodulin signaling pathways and to region-specific impairments of synaptic plasticity and cognition [8]. Although mice deficient in Ng grow with normal phenotypes, they present deficits in visual–spatial learning and a tendency towards stress and anxiety. Furthermore, they show alterations in the induction of LTP and reduced phospho-CaMKIIα levels [4].

Due to the fact that Ng plays a crucial role in processes such as synaptic plasticity, synaptic regeneration, and LTP, mediated by calcium- and calmodulin-signaling pathways, a growing body of research is investigating its role in neurodegenerative diseases. As synaptic dysfunction is thought to be an early event in the pathogenesis of neurodegenerative diseases, post-synaptic proteins, such as Ng, may have a potential as biomarkers to improve diagnosis, prognosis, and monitoring of disease progression [9]. In this review, we focus on the role of neurogranin in Alzheimer’s, Parkinson’s, and Crezutzfeldt–Jacob’s diseases.

## 2. Alzheimer’s Disease

Alzheimer’s disease is the most prevalent neurodegenerative disorder worldwide, caused by damage to nerve cells in the brain. The brain changes are thought to begin as soon as 20 years prior to onset of disease. When symptoms are severe enough to interfere with ability to perform everyday tasks, patients are said to have Alzheimer’s disease dementia [10]. The most common signs of Alzheimer’s disease dementia are as follows: memory loss disrupting everyday life, challenges in planning and solving problems, confusion with time or place, trouble understanding visual images and spatial relationships, misplacing things and losing the ability to retrace steps, withdrawal from work or social activities, as well as mood, personality, and behavioral changes. The main hallmark of AD is the accumulation of amyloid beta (Aβ) outside neurons and intraneuronal twisted strands of tau protein called neurofibrillary tangles (NFTs) [10]. Patients with AD show abnormalities in regional metabolism before brain structure changes occur. Fluorine 18 (18F) fluorodeoxyglucose (FDG) positron emission tomography (PET) is a nuclear imaging technique that indicates the metabolic activity of tissues and organs with greater sensitivity than structural magnetic resonance imaging (MRI). AD patients tend to show hypometabolism on 18F-FDG-PET scans in the regions of the posterior cingulate, parietotemporal cortices, and frontal lobes, while patients with MCI often show posterior cingulate and parietotemporal hypometabolism with variable frontal lobe involvement [11]. Compared with CSF biomarkers, topographical information on neurodegeneration obtained with 18F-FDG-PET (patterns of hypometabolism) is closely associated with the type and severity of cognitive deficits, making this particularly useful for differential diagnosis, staging of disease extent, and prediction of short-term progression [12].

Experiments on animal models indicate that synaptic dysfunction and altered synaptic plasticity are fundamental aspects underlying AD pathophysiology. It was observed that substantial synaptic reduction already occurs in individuals with prodromal mild cognitive impairment (MCI) [13]. Amyloid plaques are toxic to the brain parenchyma and induce various processes leading to synaptic failure. Studies in AD mouse models revealed that the trajectories of axons and dendrites are compromised in the proximity of amyloid plaques, thus affecting synaptic integration of signals [14].

### Neurogranin in AD

The production of Ng as well as that of other synaptic proteins is diminished in AD in both the frontal cortex and hippocampus [15]. Cerebrospinal fluid (CSF) Ng has been proposed as a potential new biomarker for synaptic degeneration and loss in the neurodegeneration (N) group from the ATN classification by the National Institute on Aging and Alzheimer’s Association (NIA-AA) research framework [16].

AD patients showed significantly higher levels of Ng compared to non-AD group (neurological acute/subacute inflammatory disorders or tumors). Furthermore, a significant correlation between CSF Ng and Mini Mental State Examination (MMSE) was found in the AD group. The authors also investigated the correlation of CSF Ng with classical AD biomarkers and revealed that the strongest positive correlation was with both total (tTau) and phosphorylated tau (pTau) in AD group [17]. In a similar study, CSF Ng was increased in AD and positively correlated with CSF tau, whereas there was a negative relationship between CSF Ng (and tau) and the CSF Aβ1-42/Aβ1-40 ratio [18]. Casaletto et al. also revealed that elevated CSF Ng was associated with higher pTau and tTau levels. It was also associated with smaller hippocampal volumes. Furthermore, there was a correlation between higher levels of CSF Ng and poorer delayed recall performances. Interestingly, Ng was the only biomarker among tTau, pTau, and Aβ1-42 significantly associated with memory scores. Given the fact that Ng is abundant in the hippocampus, a structure strongly linked to memory abilities, CSF Ng may reflect hippocampal-related changes [19]. A growing body of literature indicates the usefulness of CSF Ng in early stages of Alzheimer’s disease such as MCI. Biochemically, MCI patients present a deceleration or even a plateau in Aβ accumulation, whereas tau accumulation and further neurodegeneration continues. During this phase patients exhibit difficulties performing instrumental activities of daily living. Approximately 35–80% of MCI individuals exhibit at least one neuropsychiatric symptom, such as depression, anxiety, agitation, or sleep problems. It is estimated that 30–50% of MCI patients will convert to AD dementia over a 5–10 year period. The likelihood of progression is dependent on the risk factors, such as psychiatric disorders, metabolic syndrome, cardiovascular diseases, or the presence of at least one allele of the e4 variant of the *APOE* gene [20]. It has been shown that patients with MCI due to AD showed elevated levels of CSF Ng compared to mildly cognitively impaired subjects not diagnosed with AD. Researchers have also found increased CSF concentration of another synaptic protein, YKL-40. However, CSF Ng reached higher diagnostic accuracy compared to YKL-40 [21]. In another study, CSF Ng levels were higher in the CSF Aβ-positive MCI group, indicating that CSF Ng is an early marker of AD-related synaptic damage. Additionally, CSF Ng levels were higher among *APOE* ε4 carriers compared to non-carriers [22]. Similarly, significantly higher levels of CSF Ng were found in patients with prodromal AD (MCI patients with an AD-like CSF profile) compared to controls. There was also a strong correlation between CSF Ng and tTau as well as pTau, and a negative correlation with Aβ1-42 in the whole study population [23]. In another study, CSF Ng was increased in patients with Alzheimer’s disease dementia, progressive MCI, and stable MCI compared to controls, as well as in Alzheimer’s disease dementia and progressive MCI compared to stable MCI. In the MCI group, high baseline CSF Ng levels predicted cognitive decline, as reflected by decreased MMSE and increased Alzheimer’s Disease Assessment Scale–Cognitive Subscale scores at clinical follow-up. High baseline CSF Ng levels in the MCI group correlated with longitudinal reductions in cortical glucose metabolism and hippocampal volume at clinical follow-up. Within the progressive MCI group, elevated CSF Ng levels were associated with accelerated deterioration in Alzheimer’s Disease Assessment Scale–Cognitive Subscale. Those findings suggest that CSF Ng is already increased in prodromal AD and predicts cognitive deterioration and disease-associated changes over time [24]. Pereira et al. assessed the relationship between Aβ and tau deposition and changes in synaptic function and axonal structure over the course of AD. They revealed that the presynaptic (synaptosomal-associated protein 25 (SNAP25), growth associated protein 43 (GAP43)) and postsynaptic markers (Ng) are elevated in CSF in early Alzheimer’s disease, i.e., in Aβ-positive individuals without evidence of tau pathology. Moreover, CSF Ng was the best predictor of amyloid pathology, assessed by 18F-flutemetamol PET and 18F-flortaucipir PET, compared to the presynaptic markers SNAP25 and GAP43 which could be related to the fact that postsynaptic glutamate receptor trafficking has been shown to be a prime initial target for Aβ, thus suggesting that postsynaptic terminals might be more affected by amyloid pathology in early stages of Alzheimer’s disease [25].

Neurogranin may also have a potential in differentiation of neurodegenerative diseases. It has been found that CSF Ng concentrations were significantly higher in AD dementia compared to dementia with Lewy bodies (DLB), frontotemporal dementia (FTD), and amyotrophic lateral sclerosis (ALS). Furthermore, increased CSF Ng concentrations correlated with increased Aβ plaque load, specifically in the hippocampus and amygdala, probably reflecting synaptic damage induced by aggregation of Aβ and accumulation in plaques [26]. Similarly, AD patients showed considerably higher CSF levels of Ng than DLB/Parkinson’s disease dementia (PDD), vascular dementia (VaD), and FTD patients. Moreover, Ng correlated positively with CSF *A*β1-40 and tau. Thus, CSF Ng was able to distinguish AD dementia from non-AD dementias; however, the diagnostic accuracy was similar to CSF A*β1-42* and tau. Compared to Ng, CSF YKL-40 also differentiated AD from DLB/PDD, but not from VaD or FTD [27]. Agnello et al. revealed that AD patients had significantly higher concentrations of Ng than patients with cerebrovascular diseases, inflammatory central nervous system (CNS) diseases, and peripheral neuropathy. Moreover, Ng was moderately and inversely associated with MMSE score [28]. In another study, researchers revealed that CSF Ng, neurofilament light chain (NfL), and YKL-40 were increased in Alzheimer’s disease. However, NfL was also increased in other types of neurodegenerative diseases (FTD, CJD), suggesting that this protein is a non-disease-specific biomarker [29]. Similarly, Mattsson et al. found that CSF Ng is associated with the acceleration of cognitive decline, atrophy, and hypometabolism, primarily in the presence of Aβ pathology, whereas CSF NfL was associated with cognitive decline independent of Aβ pathology. Thus, CSF Ng and tau reflect neurodegeneration in AD, while NfL reflects neurodegeneration independent of AD [30]. On the other hand, NfL was the only one able to track the disease progression and to differentiate stages within the AD continuum. As for CSF YKL-40, its concentration showed no differences between AD dementia and FTD. CSF Ng was increased in AD dementia but reduced among FTD patients as well as patients with preclinical AD. Thus, CSF Ng seems to be more disease-specific compared to other synaptic proteins [29]. Other researchers also found increased levels of Ng in AD patients compared to patients with other neurodegenerative disorders, such as corticobasal degeneration (CBD), FTD, DLB, multiple system atrophy (MSA), Parkinson’s disease, and Pick’s dementia. Interestingly, researchers have revealed that the Aβ1-42/Ng ratio was significantly lower in an AD group and that this ratio performed better than Ng alone. More importantly, the Aβ1-42/Ng ratio showed a slightly better area under the curve (AUC) than the Aβ1-42/Aβ1-40 ratio in differentiating AD from patients with other neurodegenerative diseases. Thus, such a ratio may serve as a useful diagnostic biomarker and possibly a prognostic factor [31]. Another study investigated the role of the Ng and Aβ1-42/Ng ratio in differential diagnosis and revealed that CSF Ng concentration was higher in the AD dementia group and amnestic MCI due to AD (AD-aMCI) compared to FTDL dementia. The lowest concentration of Ng was found among MCI due to FTDL (FTLD-MCI) patients. Interestingly, Ng differentiated AD dementia from AD-aMCI with better accuracy than the Aβ1-42/Aβ1-40 ratio. Furthermore, AD-aMCI patients had significantly lower Aβ1-42/Ng ratios compared to FTDL-MCI patients. Moreover, the Aβ1-42/Ng ratio differentiated AD dementia from FTDL dementia (AUC 0.9), AD dementia from AD-aMCI (AUC 0.61), and FTDL-MCI from FTDL dementia (AUC 0.68). Thus, the use of the Aβ1-42/Ng ratio could help refine clinical approaches and target interventions more precisely to individual patients [32].

Literature data also point to the fact that CSF Ng may also serve as a biomarker reflecting disease progression. Tarawneh et al. found that CSF Ng levels predicted future cognitive impairment in controls and rates of cognitive decline in patients with symptomatic AD over time [33]. High CSF Ng levels at the MCI stage predicted progression to dementia due to AD. In amyloid-positive MCI patients, high CSF Ng correlated with a more rapid change in cognition during clinical follow-up [34]. Baseline CSF levels of Ng were also higher in patients with MCI who progressed to AD compared to those with stable MCI, and they were predictive of progression from MCI to AD. Authors disclose that CSF Ng is subject to increase at very early (i.e., asymptomatic) stages of the AD spectrum [35]. This idea fully supports the conception that synaptic alterations primarily occur during the earliest stages of AD, even before the MCI stage [36]. Headly et al. performed an 8-year follow-up study measuring CSF Ng among people with normal cognition and MCI. It turned out that an increased CSF Ng level was associated with lower baseline memory scores. Furthermore, baseline Ng levels predicted decline in memory and executive function in MCI patients over the 8-year period. These findings suggest that Ng is an important factor that plays a crucial role in memory dysfunction in AD [37]. Table 1 presents cut-off values of CSF neurogranin in Alzheimer’s disease.

## 3. Parkinson’s Disease

Parkinson’s disease (PD) is a progressive, neurodegenerative condition characterized by the loss of nigrostriatal dopaminergic neurons. The majority of PD cases are sporadic, and only 5% to 10% are genetic, caused by either autosomal dominant (e.g., *SNCA* gene encoding α-synuclein) or recessive (e.g., *PARK7* gene encoding DJ1 protein, *PINK1* gene encoding phosphatase, and tensin homolog-induced kinase 1) mutations. The key mechanisms involved in PD pathogenesis are α-synuclein production and degradation, mitochondrial dysfunction, oxidative stress, and neuroinflammation [39]. Neuropathologically, PD is characterized by the widespread aggregation of α-synuclein in the form of Lewy bodies. It has been already shown that α-synuclein oligomers and protofibrils exhibit neurotoxic effects [40]. Formation of α-synuclein-containing aggregates in neurons of the substantia nigra (Lewy bodies, Lewy neurites) leads to clinical syndromes such as bradykinesia, rigidity, tremor, and gait disturbance. Diagnosis of PD is mainly based on clinical criteria. However, changes in the brain that lead to neurodegeneration are already present several years before symptoms occurrence. This asymptomatic phase seems to be ideal to detect biochemical changes, thus enabling faster diagnosis with more effective treatment options [41].

### Neurogranin in PD

Coimmunoprecipitation studies in the superior temporal cortex in humans confirmed the interaction between α-synuclein and Ng, and a decreased interaction between α-synuclein and Ng was noticed in patients diagnosed with PD when compared to healthy control brains [42]. Ng is also found in the pyramidal neurons of the hippocampus. These areas of the cortex and hippocampus are damaged in later stages of PD, leading to the onset of dementia [43]. The degeneration of cells in areas that express Ng in Parkinson’s disease dementia indicate that Ng may be involved in pathways which lead to dementia. Additionally, Ng and α-synuclein both appear to interact with CaM in calcium signaling pathways, suggesting that Ng likely contributes a role to α-synuclein molecular functionality [42]. Recently, synaptic proteins have been considered valuable biomarkers for Parkinson’s disease, as synaptic alteration is an early event in PD. Ng levels are significantly reduced in PD patients compared to controls. Moreover, authors have investigated the value of the combination of Aβ1-42 with Ng in the form of a ratio. It turned out that the Aβ1-42/Ng ratio correlated with MMSE and differentiated cognitively impaired PD patients with 92% sensitivity, 71% specificity, and higher accuracy than the use of Ng alone. This ratio joins together presynaptic (Aβ1-42) and postsynaptic (Ng) markers, providing a global index of synaptic dysfunction with higher clinical significance that Ng alone. Aβ1-42 and Ng represent two different biomarkers of synaptopathy that are closely related to cognitive functions. Thus, the use of the Aβ1-42/Ng ratio seems like a reliable biomarker to monitor synaptic dysfunction and its correlation with cognitive performance [44]. Another group of researchers found that CSF Ng levels were significantly lower in PD patients compared to a control group. Moreover, CSF Ng correlated with concentrations of CSF Aβ1-38, Aβ1-40, Aβ1-42, and α-synuclein. Other authors also revealed that a lower CSF Ng concentration was associated with more severe reductions in FDG-PET and that there was a significant correlation between CSF Ng levels and cortical thickness among PD patients. These findings support the hypothesis that synaptic dysfunction due to altered Aβ and α-synuclein metabolism may be crucial in the evolution of cognitive impairment in PD [45]. In another study, authors also observed reduced Ng concentrations among PD patients compared to controls. Furthermore, there was a strong correlation between CSF Ng and CSF α-synuclein levels. Such reduction in Ng level is suggested to be caused by reduced synaptic activity, possibly due to presynaptic α-synuclein deposits [46]. Conversely, Bereczki et al. found that CSF Ng concentrations were notably higher among drug-naïve PD patients compared to controls. Such an increase was associated with reduced cognition and higher motor disease stage and may indicate an early-stage PD with marked postsynaptic loss, which becomes aligned as the disease progresses [47]. Table 2 presents mean values of CSF neurogranin in Parkinson’s disease. 

## 4. Creutzfeldt–Jacob’s Disease

Creutzfeldt–Jacob’s disease is the most common human prion disease, which is characterized by rapidly progressive dementia and a very short survival time. Abnormal conversion of the prion protein into pathogenic forms induces neuronal destruction, leading to spongiosis [48]. Three types of CJD may be distinguished: sporadic (sCJD), familial (fCJD), and variant (vCJD). sCJD is the most common type, accounting for about 85% of cases, and typically occurs without any known risk factors. fCJD results from genetic mutations in the *PRNP* gene encoding the prion protein. vCJD is linked to the consumption of beef products contaminated with the prion that causes bovine spongiform encephalopathy (BSE). Due to different genotype combination at codon 129 (methionine or valine) as well as pathogenic prion protein (PrP^Sc^ type 1 or 2), different subtypes may be distinguished. The most prevalent subtypes are CJDMM1/CJDMV1 (predominant cortical affection) and CJDVV2 (prominent cerebellar affection). Abnormal prion protein aggregation in synaptic structures leads to synaptic damage [49]. Typical symptoms of CJD include rapidly progressive dementia, visual disturbances, depression, anxiety, and ataxia. The diagnosis of CJD is typically based on a combination of clinical findings and test results. Electroencephalography (EEG) shows periodic sharp wave (PSW) complexes within the first 3 months of the disease in approximately 80% of cases. The presence of 14-3-3 protein and extremely high concentrations of tau protein in CSF may support the diagnosis [50].

### Neurogranin in CJD

Immunohistochemical analysis of Ng in the cerebral cortex revealed that there was a significant reduction in neural Ng along with the presence of spongiform degeneration. Authors have also revealed that Ng is not only expressed in post-synaptic compartments, but also in the neuronal body and showed positive correlation with tau protein. Such correlation may prove that CSF Ng in CJD reflects massive neuronal destruction in the brain, which occurs at early stages of CJD, similar to AD [48]. In a study by Antonell et al., CSF Ng levels were significantly higher compared to controls. Moreover, it these levels were more increased than in other neurodegenerative diseases included in the study, i.e., preclinical AD, MCI due to AD dementia, and FTD, suggesting CSF Ng’s potential role in differential diagnosis of neurodegenerative diseases [29]. Blennow et al. investigated the value of CSF Ng in CJD in comparison to AD. They found that CSF Ng was significantly higher in CJD patients than in controls (0.4 times higher). Interestingly, CSF Ng levels were not different from those evaluated at middle and late stages of disease. Those findings indicate that synaptic damage is an early event in CJD. Furthermore, CSF Ng was able to differentiate CJD from AD with great diagnostic performance (AUC 0.85). In the CJD group, the CSF concentration of Ng correlated positively with the CSF concentration of tau protein. Furthermore, patients with positive 14-3-3 tests presented higher CSF Ng levels compared to those showing no 14-3-3 signal. Thus, CSF Ng may be of diagnostic value when combined with an already established marker of CJD, such as 14-3-3 protein. As for differentiation between CJD subtypes, it was found that levels of CSF Ng were higher in CJDMM1/MV1 subtypes than in CJDVV2. This observation suggests that synaptic damage is an early event in CJD due to the fact that Ng levels did not vary significantly at early, middle, and late stage of the disease. Interestingly, compared to 14-3-3 protein, a gold-standard CSF biomarker for CJD, Ng exhibited very similar diagnostic accuracy in discriminating CJD patients from controls. Thus, Ng can reflect both synaptic and axonal damage and may be useful biomarker in early CJD diagnosis [51]. Table 3 presents mean values of CSF neurogranin in Creutzfeldt-Jacob’s disease.

## 5. Legal Aspects of the Diagnostic Value of CSF Neurogranin in ND

The ability to study neurodegenerative disease markers in CSF provides insight into neuropathological brain disorders associated with biochemical and structural changes in the central nervous system. CSF is a valuable source of proteins, although lumbar puncture is the only method of obtaining it. This is a more invasive procedure than blood sampling, and therefore, there are some guidelines regarding contraindications to lumbar puncture, as well as legal restrictions. One of these is the need for additional patient consent, which is covered by legal regulations depending on the guidelines in the countries concerned [52]. Such legal standards, specifically related to the collection of CSF in the early stages of Alzheimer’s disease, i.e., at a stage when the patient has only mild cognitive impairment, set out the conditions under which such a procedure can be performed, as the patient does not report significant complaints and the LP is performed to determine the likelihood of Alzheimer’s dementia. The risks posed by lumbar puncture are higher than those of a routine blood draw; hence, when the patient is in the final stage of AD, a period when they are not always able to give consent for additional medical procedures, including lumbar puncture, the legal regulations include guidelines for obtaining consent from the patient or their legal representative. Sometimes, there is a need to collect CSF several times. This occurs in situations where we want to monitor treatment results. It is then particularly important to develop medical law standards that define the relationship between medical professionals and their patients and that regulate the performance of clinical procedures, such as the collection of CSF by lumbar puncture. Therefore, the topic of the usefulness of neurogranin in the diagnosis of neurodegenerative diseases and the possibility of its determination in CSF, as discussed in this article, raises both medical and legal issues [52].

## 6. Conclusions

There is an urgent need for consistent biomarkers of dendritic loss such as Ng—a neuron-specific dendritic protein expressed at the post-synaptic level and mainly detected in cortical and hippocampal regions by excitatory neurons. An increasing number of studies is investigating the potential role of Ng in neurodegenerative diseases. The majority of this research focuses on Alzheimer’s disease, as synaptic degeneration is an essential component of its pathophysiology already at its earliest stages. Perturbations in synaptic integrity can be disclosed even in MCI cases, enabling early diagnosis [53]. Ng seems to also be a valuable tool in differential diagnosis, especially between Alzheimer’s disease and other neurodegenerative diseases. Furthermore, combining Ng with other well-established AD biomarkers in the form of ratios may add an additional value and increase diagnostic accuracy [27].

Although only a few studies have investigated the role of CSF Ng in Parkinson’s disease, the results are promising. Reduced CSF Ng levels seem to be a result of reduced synaptic activity due to presynaptic α-synuclein deposits, as there are strong correlations between Ng and α-synuclein levels [46]. Moreover, CSF Ng in combination with Aβ1-42 levels correlate with cognitive decline among patients with Parkinson’s disease dementia [44]. Thus, adding Ng to the pool of analyzed CSF proteins in both diagnosis and monitoring progression to dementia may be useful.

There is a lack of studies investigating CSF Ng in Creutzfeldt–Jacob’s disease. However, Blennow et al. conducted a valuable study presenting for the first time that CSF Ng may serve as a biomarker for differential diagnosis and prognosis. Moreover, Ng presented high value in distinguishing between different CJD subtypes. Therefore, further CSF Ng investigations in terms of CJD are crucial to gain more knowledge about its pathogenesis and possible use in clinical practice [51].

To conclude, fluid biomarkers reflecting synaptic dysfunction—such as Ng—are needed, given that they can be used as tools to monitor synaptic dysfunction during disease progression, therefore facilitating early diagnosis of the disease.

## Figures and Tables

**Table 1:** Cut-off values of CSF neurogranin in Alzheimer’s disease.

Cut-Off Value	Diagnostic Performance	Group	Method	References
306 pg/mL	0.84% sensitivity, 78% specificity, Youden index 0.621	AD vs. controls	ELISA	[17]
319 pg/mL	73% sensitivity, 73% specificity, AUC 0.78	AD vs. controls	ELISA	[28]
167.78 pg/mL	20% sensitivity, 85.4% specificity, AUC 0.504	AD vs. controls	ELISA	[38]
250 pg/mL	48% sensitivity, 96% specificity, AUC 0.73	controls vs. MCI+AD+FTD+CJD	ELISA	[29]
230 pg/mL	64% sensitivity, 88% specificity, AUC 0.85	healthy controls vs. AD	ELISA	[29]
293 pg/mL	68% sensitivity, 78% specificity, AUC 0.77	AD vs. non-AD disorders	ELISA	[31]
165.5 pg/mL	93% sensitivity, 41% specificity, AUC 0.699	AD vs. non-neurodegenerative disorders	ELISA	[31]

AD—Alzheimer’s disease; MCI—mild cognitive impairment; FTD—frontotemporal dementia; CJD—Creutzfeldt–Jacob’s disease; AUC—area under the curve; ELISA—enzyme-linked immunosorbent assay.

**Table 2:** Mean values of CSF neurogranin in Parkinson’s disease.

Mean Value	Group	Method	References
215.07 pg/mL vs. 336.53 pg/mL	PD vs. controls	ELISA	[44]
251 pg/mL vs. 397 pg/mL	PD vs. controls	ELISA	[45]
242.2 pg/mL vs. 311.7 pg/mL	PD vs. controls	ELISA	[46]
242.2 pg/mL vs. 480.4 pg/mL	PD vs. AD	ELISA	[46]
242.2 pg/mL vs. 339.8 pg/mL	PD vs. DLB	ELISA	[46]
359 pg/mL vs. 338 pg/mL	PD vs. controls	ELISA	[47]

PD—Parkinson’s disease; AD—Alzheimer’s disease; DLB—Lewy bodies dementia; ELISA—enzyme-linked immunosorbent assay.

**Table 3:** Mean values of CSF neurogranin in Creutzfeldt–Jacob’s disease.

Mean Value	Group	Method	References
587.84 pg/mL vs. 173 pg/mL	CJD vs. controls	ELISA	[29]
587.54 pg/mL vs. 252.4 pg/mL	CJD vs. AD	ELISA	[29]
587.54 pg/mL vs. 135.5 pg/mL	CJD vs. FTD	ELISA	[29]
571 pg/mL vs. 120 pg/mL	CJD vs. controls	ELISA	[51]
571 pg/mL vs. 233 pg/mL	CJD vs. AD	ELISA	[51]
384 pg/mL vs. 630 pg/mL	CJDVV vs. CJDMM	ELISA	[51]

CJD—Creutzfeldt–Jacob’s disease; AD—Alzheimer’s disease; FTD—frontotemporal dementia; CJDVV—VV subtype of sporadic CJD; CJDMM—MM subtype of CJD; ELISA—enzyme-linked immunosorbent assay.

## References

[1] Li H.Y., Li J.F., Lu G.W. (2003). Neurogranin: A brain-specific protein. Sheng Li Ke Xue Jin Zhan.

[2] Díez-Guerra F.J. (2010). Neurogranin, a link between calcium/calmodulin and protein kinase C signaling in synaptic plasticity. IUBMB Life.

[3] Huang K.P., Huang F.L., Chen H.C. (1993). Characterization of a 7.5-kDa protein kinase C substrate (RC3 protein, neurogranin) from rat brain. Arch. Biochem. Biophys..

[4] Zhong L., Cherry T., Bies C.E., Florence M.A., Gerges N.Z. (2009). Neurogranin enhances synaptic strength through its interaction with calmodulin. EMBO J..

[5] Garrido-García A., de Andrés R., Jiménez-Pompa A., Soriano P., Sanz-Fuentes D., Martínez-Blanco E., Díez-Guerra F.J. (2019). Neurogranin Expression Is Regulated by Synaptic Activity and Promotes Synaptogenesis in Cultured Hippocampal Neurons. Mol. Neurobiol..

[6] Wang J.H., Kelly P.T. (1995). Postsynaptic injection of Ca^2+^/CaM induces synaptic potentiation requiring CaMKII and PKC activity. Neuron.

[7] Ramakers G.M., Gerendasy D.D., de Graan P.N. (1999). Substrate phosphorylation in the protein kinase Cgamma knockout mouse. J. Biol. Chem..

[8] Mons N., Enderlin V., Jaffard R., Higueret P. (2001). Selective age-related changes in the PKC-sensitive, calmodulin-binding protein, neurogranin, in the mouse brain. J. Neurochem..

[9] Xiang Y., Xin J., Le W., Yang Y. (2020). Neurogranin: A Potential Biomarker of Neurological and Mental Diseases. Front. Aging Neurosci..

[10] (2024). 2024 Alzheimer’s disease facts and figures. Alzheimers Dement.

[11] Duan J., Liu Y., Wu H., Wang J., Chen L., Chen C.L.P. (2023). Broad learning for early diagnosis of alzheimer’s disease using fdg-pet of the brain. Front. Neurosci..

[12] Chételat G., Arbizu J., Barthel H., Garibotto V., Law I., Morbelli S., van de Giessen E., Agosta F., Barkhof F., Brooks D.J. (2020). Amyloid-PET and 18F-FDG-PET in the diagnostic investigation of Alzheimer’s disease and other dementias. Lancet Neurol..

[13] Scheff S.W., Price D.A., Schmitt F.A., DeKosky S.T., Mufson E.J. (2007). Synaptic alterations in CA1 in mild Alzheimer disease and mild cognitive impairment. Neurology.

[14] Serrano-Pozo A., Frosch M.P., Masliah E., Hyman B.T. (2011). Neuropathological alterations in Alzheimer disease. Cold Spring Harb. Perspect. Med..

[15] Davidsson P., Blennow K. (1998). Neurochemical dissection of synaptic pathology in Alzheimer’s disease. Int. Psychogeriatr..

[16] Jack C.R., Bennett D.A., Blennow K., Carrillo M.C., Dunn B., Haeberlein S.B., Holtzman D.M., Jagust W., Jessen F., Karlawish J. (2018). Contributors. NIA-AA Research Framework: Toward a biological definition of Alzheimer’s disease. Alzheimer’s Dement..

[17] Agnello L., Gambino C.M., Lo Sasso B., Bivona G., Milano S., Ciaccio A.M., Piccoli T., La Bella V., Ciaccio M. (2021). Neurogranin as a Novel Biomarker in Alzheimer’s Disease. Lab. Med..

[18] De Vos A., Jacobs D., Struyfs H., Fransen E., Andersson K., Portelius E., Andreasson U., De Surgeloose D., Hernalsteen D., Sleegers K. (2015). C-terminal neurogranin is increased in cerebrospinal fluid but unchanged in plasma in Alzheimer’s disease. Alzheimer’s Dement..

[19] Casaletto K.B., Elahi F.M., Bettcher B.M., Neuhaus J., Bendlin B.B., Asthana S., Johnson S.C., Yaffe K., Carlsson C., Blennow K. (2017). Neurogranin, a synaptic protein, is associated with memory independent of Alzheimer biomarkers. Neurology.

[20] Liss J.L., Seleri Assunção S., Cummings J., Atri A., Geldmacher D.S., Candela S.F., Devanand D.P., Fillit H.M., Susman J., Mintzer J. (2021). Practical recommendations for timely, accurate diagnosis of symptomatic Alzheimer’s disease (MCI and dementia) in primary care: A review and synthesis. J. Intern. Med..

[21] Hellwig K., Kvartsberg H., Portelius E., Andreasson U., Oberstein T.J., Lewczuk P., Blennow K., Kornhuber J., Maler J.M., Zetterberg H. (2015). Neurogranin and YKL-40: Independent markers of synaptic degeneration and neuroinflammation in Alzheimer’s disease. Alzheimer’s Res. Ther..

[22] Wang L. (2019). Alzheimer’s Disease Neuroimaging Initiative. Association of cerebrospinal fluid Neurogranin with Alzheimer’s disease. Aging Clin. Exp. Res..

[23] Sanfilippo C., Forlenza O., Zetterberg H., Blennow K. (2016). Increased neurogranin concentrations in cerebrospinal fluid of Alzheimer’s disease and in mild cognitive impairment due to AD. J. Neural Transm..

[24] Portelius E., Zetterberg H., Skillbäck T., Törnqvist U., Andreasson U., Trojanowski J.Q., Weiner M.W., Shaw L.M., Mattsson N., Blennow K. (2015). Alzheimer’s Disease Neuroimaging Initiative. Cerebrospinal fluid neurogranin: Relation to cognition and neurodegeneration in Alzheimer’s disease. Brain.

[25] Pereira J.B., Janelidze S., Ossenkoppele R., Kvartsberg H., Brinkmalm A., Mattsson-Carlgren N., Stomrud E., Smith R., Zetterberg H., Blennow K. (2021). Untangling the association of amyloid-β and tau with synaptic and axonal loss in Alzheimer’s disease. Brain.

[26] Portelius E., Olsson B., Höglund K., Cullen N.C., Kvartsberg H., Andreasson U., Zetterberg H., Sandelius Å., Shaw L.M., Lee V.M.Y. (2018). Cerebrospinal fluid neurogranin concentration in neurodegeneration: Relation to clinical phenotypes and neuropathology. Acta Neuropathol..

[27] Janelidze S., Hertze J., Zetterberg H., Landqvist Waldö M., Santillo A., Blennow K., Hansson O. (2015). Cerebrospinal fluid neurogranin and YKL-40 as biomarkers of Alzheimer’s disease. Ann. Clin. Transl. Neurol..

[28] Agnello L., Lo Sasso B., Vidali M., Scazzone C., Piccoli T., Gambino C.M., Bivona G., Giglio R.V., Ciaccio A.M., La Bella V. (2021). Neurogranin as a Reliable Biomarker for Synaptic Dysfunction in Alzheimer’s Disease. Diagnostics.

[29] Antonell A., Tort-Merino A., Ríos J., Balasa M., Borrego-Écija S., Auge J.M., Muñoz-García C., Bosch B., Falgàs N., Rami L. (2020). Synaptic, axonal damage and inflammatory cerebrospinal fluid biomarkers in neurodegenerative dementias. Alzheimer’s Dement..

[30] Mattsson N., Insel P.S., Palmqvist S., Portelius E., Zetterberg H., Weiner M., Blennow K., Hansson O. (2016). Alzheimer’s Disease Neuroimaging Initiative. Cerebrospinal fluid tau, neurogranin, and neurofilament light in Alzheimer’s disease. EMBO Mol. Med..

[31] Piccoli T., Blandino V., Maniscalco L., Matranga D., Graziano F., Guajana F., Agnello L., Lo Sasso B., Gambino C.M., Giglio R.V. (2022). Biomarkers Related to Synaptic Dysfunction to Discriminate Alzheimer’s Disease from Other Neurological Disorders. Int. J. Mol. Sci..

[32] Jurasova V., Andel R., Katonova A., Veverova K., Zuntychova T., Horakova H., Vyhnalek M., Kolarova T., Matoska V., Blennow K. (2024). CSF neurogranin levels as a biomarker in Alzheimer’s disease and frontotemporal lobar degeneration: A cross-sectional analysis. Alzheimer’s Res. Ther..

[33] Tarawneh R., D’Angelo G., Crimmins D., Herries E., Griest T., Fagan A.M., Zipfel G.J., Ladenson J.H., Morris J.C., Holtzman D.M. (2016). Diagnostic and Prognostic Utility of the Synaptic Marker Neurogranin in Alzheimer Disease. JAMA Neurol..

[34] Kvartsberg H., Duits F.H., Ingelsson M., Andreasen N., Öhrfelt A., Andersson K., Brinkmalm G., Lannfelt L., Minthon L., Hansson O. (2015). Cerebrospinal fluid levels of the synaptic protein neurogranin correlates with cognitive decline in prodromal Alzheimer’s disease. Alzheimer’s Dement..

[35] Kester M.I., Teunissen C.E., Crimmins D.L., Herries E.M., Ladenson J.H., Scheltens P., van der Flier W.M., Morris J.C., Holtzman D.M., Fagan A.M. (2015). Neurogranin as a Cerebrospinal Fluid Biomarker for Synaptic Loss in Symptomatic Alzheimer Disease. JAMA Neurol..

[36] Scheff S.W., Price D.A., Ansari M.A., Roberts K.N., Schmitt F.A., Ikonomovic M.D., Mufson E.J. (2015). Synaptic change in the posterior cingulate gyrus in the progression of Alzheimer’s disease. J. Alzheimer’s Dis..

[37] Headley A., De Leon-Benedetti A., Dong C., Levin B., Loewenstein D., Camargo C., Rundek T., Zetterberg H., Blennow K., Wright C.B. (2018). Alzheimer’s Disease Neuroimaging Initiative. Neurogranin as a predictor of memory and executive function decline in MCI patients. Neurology.

[38] Galasko D., Xiao M., Xu D., Smirnov D., Salmon D.P., Dewit N., Vanbrabant J., Jacobs D., Vanderstichele H., Eugeen V. (2019). Synaptic biomarkers in CSF aid in diagnosis, correlate with cognition and predict progression in MCI and Alzheimer’s disease. Alzheimer’s Dement..

[39] Farotti L., Paolini Paoletti F., Simoni S., Parnetti L. (2020). Unraveling Pathophysiological Mechanisms of Parkinson’s Disease: Contribution of CSF Biomarkers. Biomark. Insights.

[40] Ingelsson M. (2016). Alpha-Synuclein Oligomers-Neurotoxic Molecules in Parkinson’s Disease and Other Lewy Body Disorders. Front. Neurosci..

[41] Morris H.R., Spillantini M.G., Sue C.M., Williams-Gray C.H. (2024). The pathogenesis of Parkinson’s disease. Lancet.

[42] Koob A.O., Shaked G.M., Bender A., Bisquertt A., Rockenstein E., Masliah E. (2014). Neurogranin binds α-synuclein in the human superior temporal cortex and interaction is decreased in Parkinson’s disease. Brain Res..

[43] McKeith I.G., Boeve B.F., Dickson D.W., Halliday G., Taylor J.P., Weintraub D., Aarsland D., Galvin J., Attems J., Ballard C.G. (2017). Diagnosis and management of dementia with Lewy bodies: Fourth consensus report of the DLB Consortium. Neurology.

[44] Sancesario G.M., Di Lazzaro G., Alwardat M., Biticchi B., Basile V., Salimei C., Colona V.L., Sinibaldi Salimei P., Bernardini S., Mercuri N.B. (2020). Amyloid-β42/Neurogranin Ratio as a Potential Index for Cognitive Impairment in Parkinson’s Disease. J. Alzheimer’s Dis..

[45] Selnes P., Stav A.L., Johansen K.K., Bjørnerud A., Coello C., Auning E., Kalheim L., Almdahl I.S., Hessen E., Zetterberg H. (2017). Impaired synaptic function is linked to cognition in Parkinson’s disease. Ann. Clin. Transl. Neurol..

[46] Janelidze S., Zetterberg H., Brix B., Mattsson N., Surova Y., Blennow K., Hansson O. (2020). Cerebrospinal fluid levels of neurogranin in Parkinsonian disorders. Mov. Disord..

[47] Bereczki E., Bogstedt A., Höglund K., Tsitsi P., Brodin L., Ballard C., Svenningsson P., Aarsland D. (2017). Synaptic proteins in CSF relate to Parkinson’s disease stage markers. NPJ Park. Dis..

[48] Villar-Pique A., Zerr I., Llorens F. (2020). Cerebrospinal fluid neurogranin as a new player in prion disease diagnosis and prognosis. Neural Regen. Res..

[49] Gambetti P., Kong Q., Zou W., Parchi P., Chen S.G. (2003). Sporadic and familial CJD: Classification and characterisation. Br. Med. Bull..

[50] Wang P.S., Wu Y.T., Hung C.I., Kwan S.Y., Teng S., Soong B.W. (2008). Early detection of periodic sharp wave complexes on EEG by independent component analysis in patients with Creutzfeldt-Jakob disease. J. Clin. Neurophysiol..

[51] Blennow K., Diaz-Lucena D., Zetterberg H., Villar-Pique A., Karch A., Vidal E., Hermann P., Schmitz M., Ferrer Abizanda I., Zerr I. (2019). Llorens FCSF neurogranin as a neuronal damage marker in CJD: A comparative study with AD. J. Neurol. Neurosurg. Psychiatry.

[52] Guzik-Makaruk E.M., Pływaczewski E.W., Laskowska K., Filipkowski W., Jurgielewicz-Delegacz E., Mroczko P. (2019). A Comparative Analysis of the Treatment of Decision-Making by or for Patients with Neurodegenerative Diseases in Four Legal Jurisdictions. J. Alzheimer’s Dis..

[53] Santos A.N., Ewers M., Minthon L., Simm A., Silber R.-E., Blennow K., Prvulovic D., Hansson O., Hampel H. (2006). Amyloid-β oligomers in cerebrospinal fluid are associated with cognitive decline in patients with Alzheimer’s disease. J Alzheimers Dis 2012;29:171-6. and Scheff SW, Price DA, Schmitt FA, Mufson EJ. Hippocampal synaptic loss in early Alzheimer’s disease and mild cognitive impairment. Neurobiol. Aging.

